# Orbital Tumors Excision without Bony Marginotomy under Local and General Anesthesia

**DOI:** 10.1155/2014/424852

**Published:** 2014-04-14

**Authors:** Robert A. Goldberg, Daniel B. Rootman, Nariman Nassiri, David B. Samimi, Joseph M. Shadpour

**Affiliations:** Division of Orbital and Ophthalmic Plastic Surgery, Jules Stein Eye Institute, David Geffen School of Medicine, University of California at Los Angeles, 100 Stein Plaza, Los Angeles, CA 90095, USA

## Abstract

To present our experience of removing middle to deep orbital tumors using a combination of minimally invasive soft tissue approaches, sometimes under local anesthesia. * Methods.* In this retrospective case series, 30 patients (13 males and 17 females) underwent tumor removal through eyelid crease (17 eyes), conjunctival (nine eyes), lateral canthal (two eyes), and transcaruncular (two eyes) approaches. All tumors were located in the posterior half of the orbit. Six cases were removed under monitored anesthesia care with local block, and 24 were under general anesthesia. * Results.* The median (range) age and follow-up duration were 48.5 (31–87) years old and 24.5 (4–375) weeks, respectively. Visual acuity and ocular motility showed improvement or no significant change in all but one patient at the latest followup. Confirmed pathologies revealed cavernous hemangioma (15 cases), pleomorphic adenoma (5 cases), solitary fibrous tumor (4 cases), neurofibroma (2 cases), schwannoma (2 cases), and orbital varix (1 case). None of the patients experienced recurrence. * Conclusions.* Creating a bony marginotomy increases intraoperative exposure of the deep orbit but adds substantial time and morbidity. Benign orbital tumors can often be removed safely through small soft-tissue incisions, without bone removal and under local anesthesia.

## 1. Introduction 

Many benign tumors can affect the orbit and, if symptomatic or cosmetically disfiguring, most can be removed via various cutaneous and bony approaches. Many of these approaches involve removal of the lateral orbital wall, with replacement after tumor excision [1–7]. This approach can produce excellent operative exposure of the lesion and allow for removal with minimal manipulation of the orbital contents. With interest in minimizing surgical risk, postoperative recovery and scarring, recent surgical trends favor minimally invasive techniques and local anesthesia where possible [1, 2].

Traditionally, orbital tumors located in the middle to posterior orbit are approached from a variety of soft-tissue incisions incorporating a bony lateral orbitotomy (marginotomy) of the zygoma [1–7]. The bony flap increases exposure of the deep orbit and provides the surgeon with added maneuverability for tumor removal [6, 7]. However, the bony marginotomy can be associated with longer operative time, increased postoperative pain, recovery time, temporalis muscle wasting, and external scar [6]. Using modern imaging modalities in conjunction with minimal incision surgical techniques, we have found that bony marginotomy is rarely needed in order to access presumed benign tumors of the middle and deep orbit. In this study, we present our surgical experience in the removal of orbital tumors using a combination of soft-tissue approaches, without bony marginotomy, under monitored local anesthesia and general anesthesia.

## 2. Methods 

### 2.1. Study Subjects

In this retrospective case series, electronic medical records of all patients with orbital tumors removed through the Oculoplastics Clinic of the Jules Stein Eye Institute between 1992 and 2013 were reviewed. All patients with tumors removed without bony marginotomy were included in the study. The Institutional Review Board at the University of California, Los Angeles approved the study protocol, and the tenets of the Declaration of Helsinki were followed.

### 2.2. Outcome Measures

Data including snellen visual acuity, ocular motility, tumor size and type, follow-up duration, recurrence, and intra- and postoperative complications were recorded. Tumor size was calculated measuring longest dimension on preoperative CT or MRI. Imaging was used to classify tumor location as posterior or anterior based on confinement to the anterior half of the orbit or extension into the posterior half, respectively. Tumor or tissue types were obtained from pathology reports.

### 2.3. Surgical Techniques

**Figure 1:**
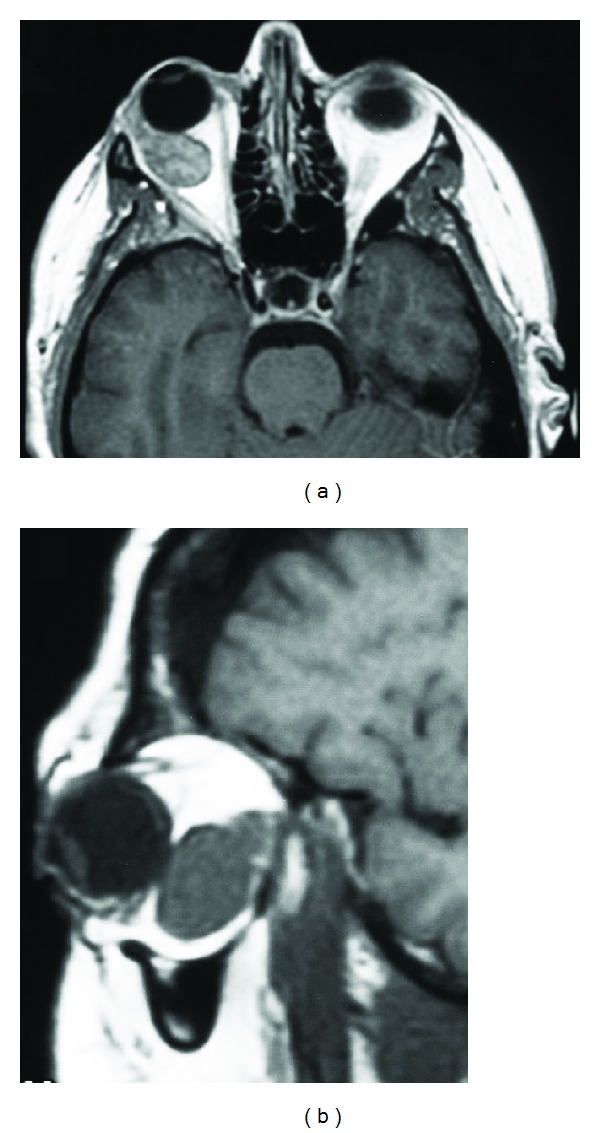
Magnetic resonance imaging of a 49-year-old female 6 weeks prior to removal of a right sided 22.9 mm cavernous hemangioma.

**Figure 2:**
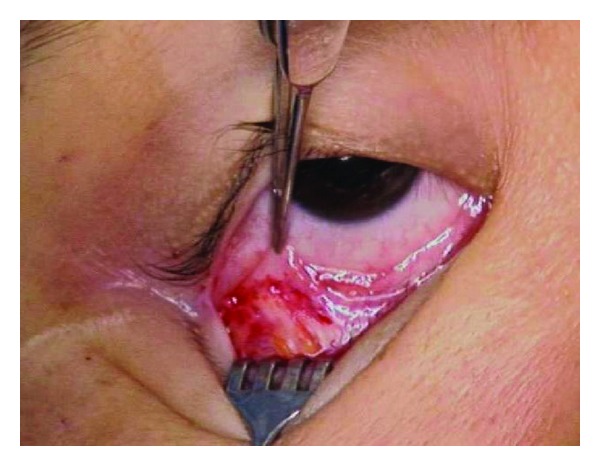
Initial inferior fornix conjunctival incision.

In most cases, surgeries were performed under general anesthesia. However, if the case was amenable to completion under local anesthesia, an array of orbital and regional nerve blocks was utilized [8]. After adequate anesthesia is achieved, the appropriate incision is chosen based on location of the tumor within the orbit. Tumors inferior to the optic nerve are generally approached with a conjunctival incision 4 mm below the inferior tarsal margin through the lower eyelid retractors and into orbital fat (Figures 1 and 2). The incision can be extended medially or laterally as necessary. When extending the incision laterally, the lateral canthal tendon can be loosened slightly by blunt spreading with scissors, avoiding complete release from the orbital rim.

For tumors located superiorly, an incision is made through the upper eyelid crease 8–10 mm above the lid margin. The incisions were customized medially or laterally based on the location of tumor and the site needs to be exposed. Vertical spread of the orbicularis fibers reveals the septum blending with the levator fibers. An incision through the septum with medial or lateral displacement of the levator fibers allows blunt dissection through the intermuscular septum. The levator can be safely distracted medially or laterally and we have not found a vertical lid splitting procedure necessary to access the superior orbit.

Working towards the anticipated 3-dimensional location of the tumor predicted by imaging studies, blunt dissection is utilized to find the anterior tip of the tumor. Balloting with paired malleable retractors can facilitate this step. Stevens scissors and peanut or cotton tipped applicators can be used to dissect fatty soft tissue off the face of the tumor. Once the face of the tumor is within view, the surgeon can assess the gross surgical pathology and confirm the diagnosis of a benign lesion.

**Figure 3:**
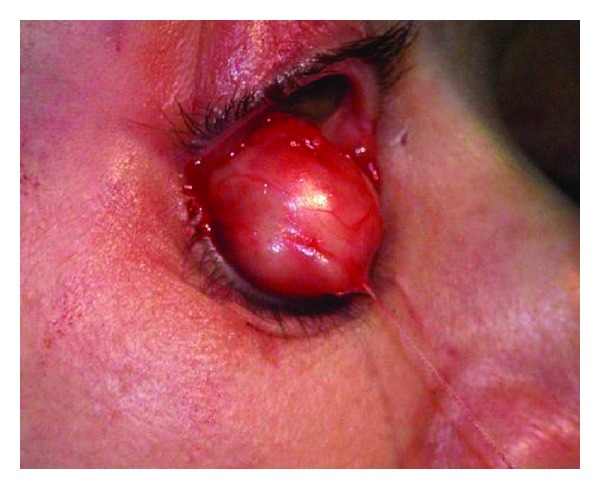
Right sided 28 mm schwannoma expressed through anterior traction by the whip suture.

**Figure 4:**
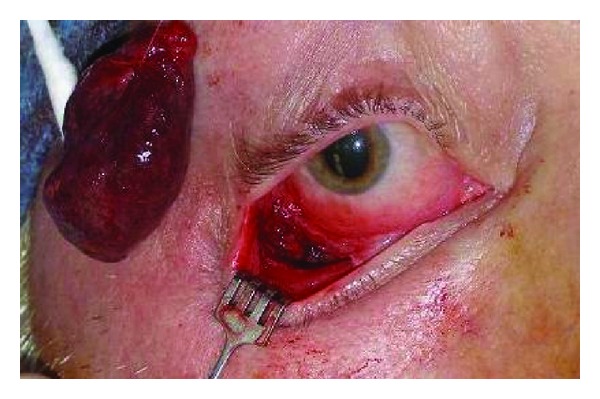
Exsanguinated right sided 34 mm cavernous hemangioma after removal through conjunctival incision under local anesthesia.

A half-circle needle can now be passed through the face of the tumor if appropriate, which if compressible will cause partial exsanguination and a decrease in tumor size. An additional pass of the needle is utilized to create a whip suture that will provide better control and forward traction. In the case of cavernous malformations, this stitch also aids in exsanguination of the lesion, decreasing its overall size for delivery anteriorly. Some tumors such as dermoid cysts or schwannomas can be additionally decompressed by suctioning the contents through a small anterior incision into the tumor. With the tumor partly exsanguinated or decompressed (if possible) and suture in place, blunt dissection (e.g., with a Freer elevator) can now be carried back toward the posterior edge of the tumor with simultaneous forward traction. As the attachments are dissected, the tumor will move towards the surgical opening, revealing posterior attachments, which can be gently lysed with blunt and sharp dissection (Figure 3). There are usually no major vascular stalks associated with cavernous hemangiomas, but any bleeding vessels are controlled with bipolar cautery (Figure 4).

**Figure 5:**
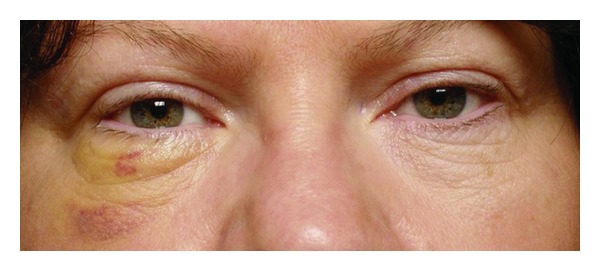
A forty-nine-year-old female 6 days after surgery.

The patient is typically able to go home the same day. Small conjunctival or eyelid crease incisions allow a relatively quick recovery with minimal ecchymosis and minimal visible scar (Figure 5).

## 3. Results

Thirty cases (13 male and 17 female) were identified. Median age was 48.5 (range 31–87) years old and median follow-up was 24.5 (range 4–375) weeks (Table 1). CT or MRI records were available for all patients. The average ± standard deviation tumor size, measured in its longest dimension based on preoperative imaging, was 22.6 ± 6.5 mm (range 10–51). Twenty-nine (out of 30) tumors were located in the posterior half of the orbit. Surgeries were performed through eyelid crease (17 eyes), conjunctival (9 eyes), lateral canthal (2 eyes), and transcaruncular (2eyes) approaches. Six cases were performed with monitored anesthesia care and local block, and 24 were performed under general anesthesia.

There were no severe intra- or postoperative surgical complications. None of the patients experienced recurrence. At the last follow-up visit, visual acuity and ocular motility showed improvement or no significant change in all patients. Two patients (# 16&29) had mild decrease in visual acuity at the last follow-up, which was related to ocular surface changes. There were no cases of new or worsened optic neuropathy. Confirmed surgical pathology revealed several tumor types including 15 cavernous hemangiomas, 5 pleomorphic adenomas, 4 solitary fibrous tumors, 2 neurofibro- mas, 2 schwannomas, and 1 orbital varix.

## 4. Discussion

Middle to deep orbital tumors are most commonly removed through a bony lateral marginotomy [1–7]. This technique was first described by Kronlein in 1889 for the removal of dermoid cysts, providing a relatively wide field of view to search for retrobulbar tumors [9]. With the advent of modern imaging modalities and ability to distinguish tumor character and location preoperatively, other less invasive soft-tissue approaches have been described.

All documented series have reported surgical excision under general anesthesia and typically involve a lateral canthotomy or rectus resection for exposure. Both Kiratli et al. and Cheng et al. presented series of intraconal orbital cavernous hemangiomas removed via transconjunctival approach [10, 11]. Their series were limited to tumors touching or near the globe and necessitated removal of the medial rectus for larger tumors. In addition, all patients in their series were operated on under general anesthesia. Another 2004 paper by Yan and Wu presented results of removing 139 (out of 214) orbital cavernomas through an anterior orbitotomy [12]. A skin incision was performed in 69 cases and conjunctival incision in 70 [12]. The remaining 75 tumors (out of 214) were removed successfully by standard lateral orbitotomy with bony marginotomy. The authors stated that traditional lateral orbitotomy must be used in cases where tumor dimensions exceed 3 cm, or the tumor extends to the orbital apex, or has imaging inconsistent with cavernous hemangioma [12]. In another series limited to cavernous hemangiomas, Pelton and Patel excised 5 medial intraconal cavernomas through a superomedial eyelid crease [13]. The authors concluded that the superomedial lid crease approach to the medial intraconal space has a number of advantages over the medial transconjunctival and lateral orbital approaches, including ease of dissection, incision-to-nerve distance, and angle of approach to the optic nerve [13].

With appropriate screening and intraoperative flexibility, we believe the most benign intraconal tumors can be removed through one of many soft-tissue approaches, often under local anesthesia. If the lesion is compressible, for example, cavernous hemangioma or dermoid cyst, large size is not a contraindication to a small incision approach. Several factors influence the patient's candidacy for minimally invasive, soft-tissue surgery. The anticipated pathology should be consistent with a benign tumor. Additionally CT or MRI imaging modalities should demonstrate a well-defined mass, without tethering, infiltration into surrounding tissue or growth into bone or the sinuses [10]. These features suggest that the tumor is likely benign in nature and distinct from the surrounding tissues making it possible to be completely resected.

Location of the tumor guides incisional approach. Tumors located in the inferior orbit are best approached through an inferior conjunctival incision. Most often a canthotomy is not required, but for more exposure, the canthus can be loosened with a small internal incision, leaving the skin and orbicularis intact. This minimal canthotomy will heal by intention, so that no suture is required. Occasionally a deep medial orbital tumor adjacent to the periosteum may be more easily accessed using a caruncular incision. Superiorly positioned orbital tumors are removed through an upper eyelid crease incision with appropriate medial or lateral orbital entry (working around the levator aponeurosis). Most benign tumors of the deep orbit can be safely reached through careful dissection with any of these incisional approaches, although tumors at the deepest apex, for example, those extending through the superior orbital fissure, generally require bone removal for optimal exposure.

The surgeon must maintain a 3-dimensional sense of the tumor in the orbit. This is achieved through careful examination of fine cut orbital CT or MRI images preoperatively in combination with adequate lighting and the use of intraoperative landmarks such as the bony rim, globe, and rectus muscles. Using this spatial knowledge along with palpation and ballottement of the tumor during dissection allows the surgeon to accurately focus the blunt and sharp dissection through the relatively small opening. Once the dissection has reached the face of the tumor, the gross pathologic appearance should be assessed. If visual inspection and palpation suggest an infiltrative process, the surgeon must be flexible enough to change the operative plan, for example, to biopsy the lesion or convert to a larger access procedure under general anesthesia. None of our cases necessitated conversion from monitored anesthesia care to general anesthesia or bone removal. The next critical point is the pass of the half-circle needle through the face of the tumor. In the case of a cavernous venous malformation (cavernous hemangioma), the resulting exsanguination of the tumor, which can be easily suctioned, shrinks the tumor in size and allows more space to maneuver. Additional passes of the needle create a whip suture, allowing forward traction for adequate dissection and expression of the tumor through the small soft-tissue incision.

Local anesthesia is preferred by many patients as it shortens the operative time, avoids the discomfort and risks of general anesthesia, and allows the patient to return home sooner. We have found that benign orbital tumors can be removed under monitored local anesthesia in some patients. Patient selection is important for successful local anesthesia orbital surgery. Patients who are overly anxious will do better under general anesthesia although moderate anxiety will respond to common anxiolytic agents used for monitored anesthesia [6, 7]. In our series, those who underwent monitored anesthesia did well with common anxiolytic agents. Intraoperatively, the orbit should become adequately anesthetized superficially and deep with blocks of the zygomaticotemporal, zygomaticofacial, infraorbital, and supraorbital nerves as well as along the orbital floor back to the level of the superior orbital fissure. It is important to maintain communication with the patient and provide more local anesthetic when necessary. More local anesthetic is often necessary in the central and medial orbit when dissecting down to the orbital rim, as there are many sensory nerves in this region. The retrobulbar block may dampen or eliminate the physiologic pupillary reaction of the ipsilateral eye. This should not affect operative decision-making: the surgeon always uses maximal care to minimize traction and pressure on nerves in the orbit. Pupillary dilation is more often related to the efferent nerves to the pupil and is not a reliable sign of optic nerve compression. Optic nerve function can be better assessed, if needed, by looking for a reverse afferent papillary defect in the opposite pupil.

The small incision approaches place an increased premium on the preoperative evaluation. This includes a detailed history, physical exam, and careful study of appropriate imaging modalities. Combining this data with a thoughtful orbital differential diagnosis, appreciation for nuances of intraoperative gross surgical pathology, and readiness to convert to an open procedure if necessary allows the surgeon to safely approach most orbital tumors with the techniques described above.

There is no rote approach to orbital tumor excision surgery. The desire to perform minimally invasive surgery should not be pursued to the extent that adequate exposure or patient safety is compromised in any way. However, within the constraints of good judgment and safety, it is appropriate to try to minimize the invasiveness of orbital surgery. In our experience, with appropriate preoperative evaluation and a creative flexible surgical approach, many benign orbital tumors can be safely approached through a minimally invasive soft-tissue approach, avoiding a bony marginotomy.

## Figures and Tables

**Table 1:** Results of orbital tumor excisions without bony marginotomy.

Patient	Anesthetic	Side	Approach	Pathology	Sex	Age	Length (mm)	Orbital depth	Preoperative visual acuity	Postoperative visual acuity	Follow-up time (weeks)
1	General	R	Conj	CH	M	47	22	P	20/20	20/20	45
2	General	R	Conj	CH	F	33	16	P	20/50	20/40	29
3	MAC	L	Conj	CH	F	48	25	P	20/25	20/15	14
4	MAC	R	Conj	CH	F	38	27	P	20/20	20/50	13
5	General	R	Conj	CH	F	82	20	P	20/30	20/25	9
6	MAC	R	Conj	CH	F	61	15	P	20/30	20/25	70
7	MAC	R	Conj	CH	F	47	31	P	20/20	20/25	111
8	MAC	R	Conj	CH	M	60	39	P	20/25	20/30	133
9	General	R	EC	CH	F	72	17	P	20/25	20/20	39
10	General	R	EC	CH	M	32	35	P	20/20	20/20	97
11	General	R	EC	CH	F	41	20	P	20/20	20/20	15
12	General	R	LC	CH	F	74	23	P	20/25	20/20	15
13	General	R	LC	CH	F	36	14	P	CF	20/20	135
14	General	L	TC	CH	F	35	17	P	20/20	20/20	305
15	General	R	TC	CH	F	56	17	P	CF	20/20	375
16	General	R	EC	Neurofibroma	M	49	15	P	20/40	20/70	13
17	General	R	EC	Neurofibroma	M	35	12	P	20/20	20/20	5
18	General	L	EC	Orbital varix	F	54	14	P	20/20	20/25	15
19	General	R	EC	PA	F	38	22	P	20/20	20/20	18
20	General	R	EC	PA	M	31	27	P	20/20	20/20	1
21	General	R	EC	PA	M	69	25	P	20/60	20/40	298
22	General	R	EC	PA	M	44	25	P	20/20	20/20	173
23	General	R	EC	PA	M	31	27	P	20/20	20/20	1
24	MAC	R	Conj	Schwannoma	F	49	28	P	20/40	20/25	49
25	General	R	EC	Schwannoma	M	41	18	P	20/20	20/20	161
26	General	L	EC	SF	M	79	26	P	20/60	20/20	58
27	General	L	EC	SF	M	61	29	P	20/20	20/20	17
28	General	L	EC	SF	M	65	24	P	20/20	20/20	8
29	General	L	EC	SF	F	51	25	A	20/50	20/100	1
30	General	L	EC	SF	F	87	20	P	CF	20/400	20

R: right; L: left; A: anterior, P: posterior, CH: cavernous hemangioma, PA: pleomorphic adenoma, SF: solitary fibrous tumor, CF: counting finger; Conj: conjunctival approach for tumors inferior to the optic nerve; TC: transcaruncular approach for deep medial orbital tumors adjacent to the periosteum; EC: eyelid crease for tumors locating superiorly; LC: lateral canthal approach for tumors locating laterally.
